# Supplementary material for: Evaluating the reliability of tools for mRNA annotation and IRES studies
Source: bioRxiv. 2026 Mar 31:2026.03.29.707813. Preprint. [Version 1] doi: 10.64898/2026.03.29.707813 (PMC13060157; doi:10.64898/2026.03.29.707813)
Supplement: Supplement 6 [file NIHPP2026.03.29.707813v1-supplement-6.pdf]

bioRxiv preprint doi: https://doi.org/10.64898/2026.03.29.707813; this version posted March 31, 2026. The copyright holder for this preprint (which was not certified by peer review) is the author/funder, who has granted bioRxiv a license to display the preprint in perpetuity. It is made available under aCC-BY 4.0 International license.

Supplemental Figures:

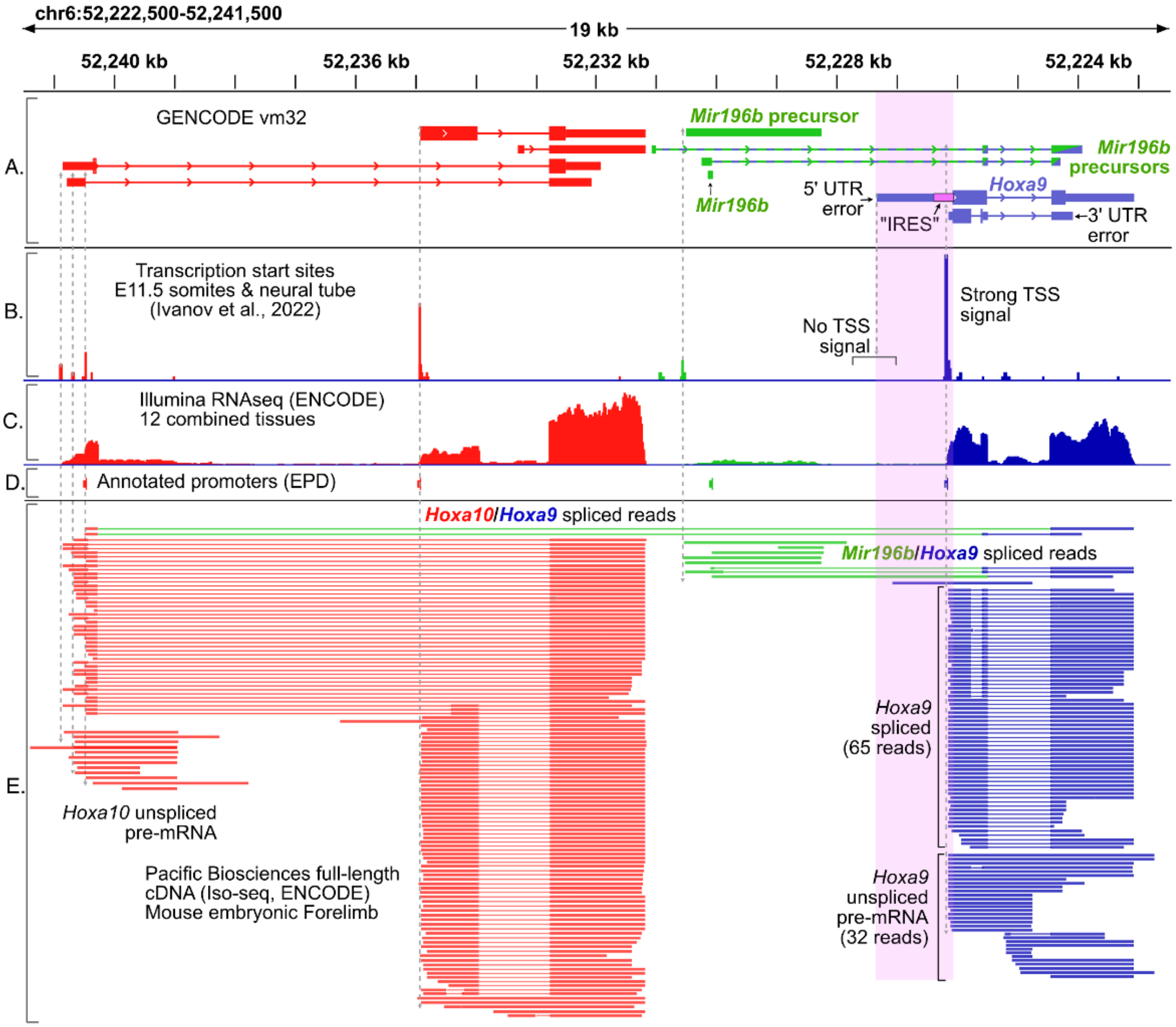

Figure S1. RNA-seq from mouse embryos shows transcription from Hoxa10 and miR196b extending through the Hoxa9 promoter and coding region. Genome browser tracks showing the Hoxa10, Mir196b, and Hoxa9 loci in the mouse genome (mm10). The genomic coordinates have been oriented to show the region from 5' to 3'. **(A)** GENCODE (version m32) transcript annotations are shown, including Hoxa10 (red), Mir196b (green), precursor transcripts for mir196b spliced to Hoxa9 (green / blue), and Hoxa9 (blue). The two Hoxa9 isoforms are misannotated due to errors in their 5' and 3' UTR coordinates. **(B)** nAnTi-CAGE sequencing data mapping m7G-capped 5' transcription start sites (TSS) are plotted (Ivanov et al., 2022), showing the major transcription start sites (dashed gray arrows). Note the total absence of transcription start site data at the misannotated "IRES" isoform of Hoxa9. Illumina **(C)** RNA-seq data combined from twelve mouse tissues (Akirtava et al. 2022). The RNA-seq signal increases immediately downstream of the somite and neural tube Hoxa9 transcription start site, with scant mRNA signal upstream in the 5' UTR of the "IRES" isoform. **(D)** Eukaryotic promoter database promoter annotations. **(E)** PacBio Iso-Seq full-length cDNA reads from mouse embryonic forelimb are shown. Two reads show Hoxa10 - Hoxa9 fusion transcripts generated by intron splicing. Multiple reads correspond to Mir196b precursor transcripts (green), including Mir196b transcripts spliced to Hoxa9 sequences (green / blue). The misannotated Hoxa9 5' UTR and promoter region overlaps pri-mir196b precursor transcripts and introns from Hoxa10/Hoxa9 fusion transcripts and precursor transcripts from Mir196b (pink rectangle). Adapted from Akirtava et al., 2022.

bioRxiv preprint doi: https://doi.org/10.64898/2026.03.29.707813; this version posted March 31, 2026. The copyright holder for this preprint (which was not certified by peer review) is the author/funder, who has granted bioRxiv a license to display the preprint in perpetuity. It is made available under aCC-BY 4.0 International license.

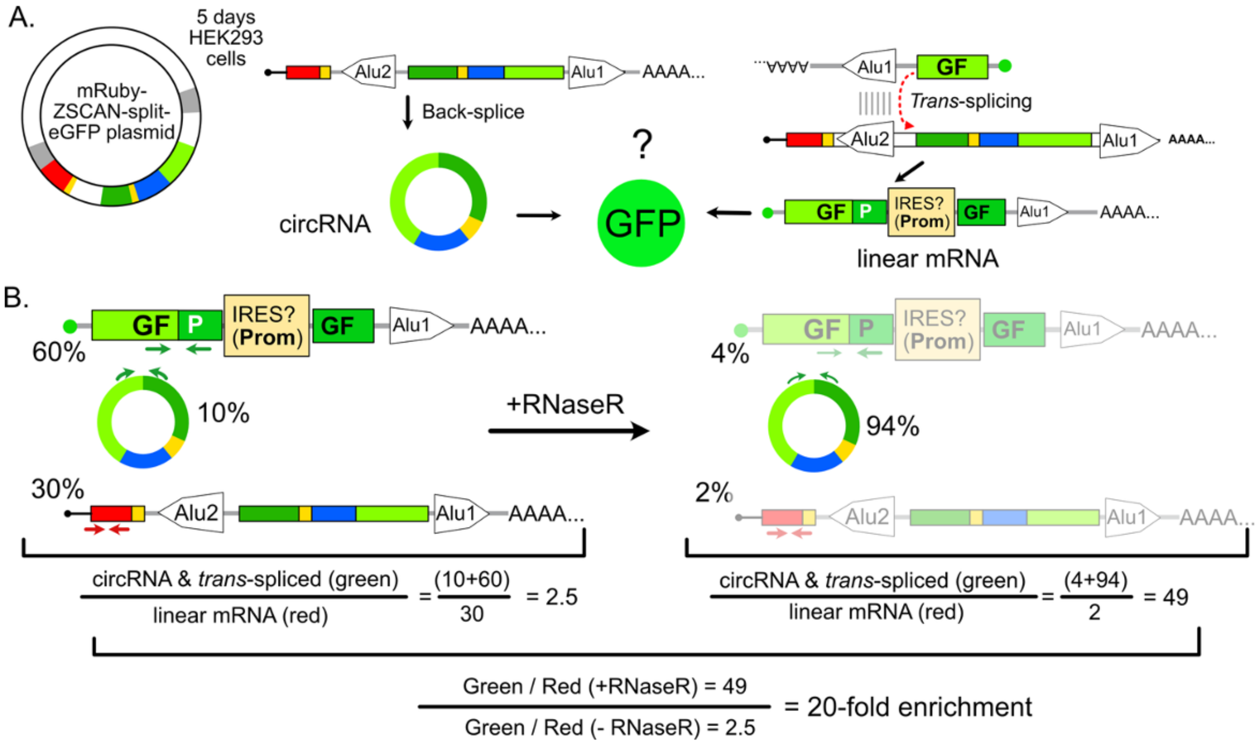

Figure S2. Enrichment of spliced GFP signal vs RFP after RNase R treatment does not rule out the presence of *trans*-spliced linear GFP mRNA. (**A**) Diagram depicts the circular RNA products produced by back-splicing (left) and the monocistronic linear GFP mRNA artifacts products produced by *trans*-splicing (right). CircRNA can produce GFP if the insert sequence (blue) has IRES activity. The linear mRNA produced by *trans*-splicing could produce GFP by cap-dependent scanning. If there are any *trans*-spliced artifacts, it is impossible to know whether GFP is expressed due to IRES activity or due to cap-dependent scanning. (**B**) The qRT-PCR assay that Koch et al. used in attempts to show GFP is expressed from circRNAs used primers across the GFP splice junction (green) and in mRuby (red). GFP primers amplify both *trans*-spliced contaminants and circRNA, while mRuby primers amplify only the linear pre-mRNA. RNase R treatment selectively depletes both the linear mRuby and the linear *trans*-spliced monocistronic GFP mRNAs. The result is an enrichment of signal from the GFP amplicon, even when *trans*-spliced linear contaminants are more abundant than circRNA. The assay only indicates that circular RNAs were present. It does not rule out the presence of *trans*-spliced contaminant artifacts.
